# Sensitive poliovirus detection using nested PCR and nanopore sequencing: a prospective validation study

**DOI:** 10.1038/s41564-023-01453-4

**Published:** 2023-08-17

**Authors:** Alexander G. Shaw, Tresor Kabeya Mampuela, Emmanuel Lokilo Lofiko, Catherine Pratt, Catherine Troman, Erika Bujaki, Áine O’Toole, Joyce Odeke Akello, Adrienne Amuri Aziza, Eddy Kinganda Lusamaki, Jean Claude Makangara, Marceline Akonga, Yvonne Lay, Bibiche Nsunda, Bailey White, David Jorgensen, Elizabeth Pukuta, Yogolelo Riziki, Kathleen E. Rankin, Andrew Rambaut, Steve Ahuka-Mundeke, Jean-Jacques Muyembe, Javier Martin, Nicholas C. Grassly, Placide Mbala-Kingebeni

**Affiliations:** 1 MRC Centre for Global Infectious Disease Analysis, School of Public Health, Imperial College London, London, UK; 2 Service de Microbiologie, Departement de Biologie Médicale, Cliniques Universitaires de Kinshasa (CUK), Université de Kinshasa (UNIKIN), Kinshasa, Democratic Republic of the Congo; 3 Institut National de Recherche Biomédicale, Kinshasa, Democratic Republic of the Congo; 4 College of Public Health, University of Nebraska Medical Center, Omaha, NE USA; 5 Department of Vaccines, National Institute for Biological Standards and Control (NIBSC), Medicines and Healthcare products Regulatory Agency, Potters Bar, UK; 6 Institute of Ecology and Evolution, University of Edinburgh, Ashworth Laboratories, Edinburgh, UK; 7 TransVIHMI (Recherches Translationnelles sur le VIH et les Maladies Infectieuses endémiques et émergentes), University of Montpellier (UM), French National Research Institute for Sustainable Development (IRD), INSERM, Montpellier, France; 8 Bill and Melinda Gates Foundation, Seattle, WA USA

**Keywords:** Translational research, Sequencing

## Abstract

Timely detection of outbreaks is needed for poliovirus eradication, but gold standard detection in the Democratic Republic of the Congo takes 30 days (median). Direct molecular detection and nanopore sequencing (DDNS) of poliovirus in stool samples is a promising fast method. Here we report prospective testing of stool samples from suspected polio cases, and their contacts, in the Democratic Republic of the Congo between 10 August 2021 and 4 February 2022. DDNS detected polioviruses in 62/2,339 (2.7%) of samples, while gold standard combination of cell culture, quantitative PCR and Sanger sequencing detected polioviruses in 51/2,339 (2.2%) of the same samples. DDNS provided case confirmation in 7 days (median) in routine surveillance conditions. DDNS enabled confirmation of three serotype 2 circulating vaccine-derived poliovirus outbreaks 23 days (mean) earlier (range 6–30 days) than the gold standard method. The mean sequence similarity between sequences obtained by the two methods was 99.98%. Our data confirm the feasibility of implementing DDNS in a national poliovirus laboratory.

## Abstract (web summary)

Prospective validation in the Democratic Republic of the Congo shows that implementation of nested polymerase chain reaction and nanopore sequencing is feasible in a national poliovirus laboratory

## Main

Despite substantial progress made by the Global Polio Eradication Initiative (GPEI), since inception in 1988, poliomyelitis remains a major public health problem in countries with low vaccination coverage. Mass vaccination campaigns with oral poliovirus vaccine (OPV) are used during poliovirus outbreaks to stop transmission. However, a combination of slow shipping of stool samples, time-consuming virus isolation using cell culture and insufficient sequencing capacity delay outbreak responses and reduce the impact of mass vaccination campaigns^1–3^.

In August 2020, the African Region was declared to have interrupted the transmission of wild poliovirus (WPV)^4^. Vaccination with OPV has resulted in outbreaks of circulating vaccine-derived poliovirus (cVDPV), which occurs by reversion of attenuating mutations in the live-vaccine strain. Live-vaccine strains are shed in faeces following vaccination and can spread in under-immunized populations, with the attenuating mutations being lost over time^5,6^. Serotype 2 cVDPV (cVDPV2) epidemics in young children plague Africa, and Western Asia, in this post-WPV era. In 2020, 959 cases of paralysis caused by cVDPV2 were reported in 27 countries, including 21 countries in Africa^7^; in 2021, 692 cases caused by cVDPV2 and 20 cases by serotype 1 cVDPV were reported globally, mainly in Africa including Nigeria and the Democratic Republic of the Congo (DRC)^8^. In 2022 at least 843 cases of VDPV were reported, 502 of which were in the DRC^8^.

In DRC, 10 years after the last case of WPV, there has been almost continual circulation of cVDPV2 as a result of emergence of cVDPV2 following the use of serotype 2 OPV in response to existing outbreaks. Responses are hampered by inadequate surveillance and lengthy times before outbreaks are confirmed. Poliovirus surveillance is based on the collection of stool samples from children with acute flaccid paralysis (AFP) and their contacts, and on environmental (sewage) sampling. Effective poliovirus surveillance relies on high-quality sample collection and laboratory testing. Stool collected from an AFP case is considered adequate for testing if two stools are collected 48 h apart, within 2 weeks of onset of paralysis, and arrive by cold chain with proper documentation. In DRC, the proportion of AFP cases with inadequate stool sample collection was 23% in 2018 (ref. ^8^). Additionally, logistical challenges in sample shipment to the laboratory, in laboratory testing of the samples and in international shipment (to South Africa) for sequencing cause delayed detection of poliovirus outbreaks. Case numbers from an outbreak have been estimated to increase by approximately 12% (95% credible interval 5–21%) per additional week^9^ (average of data for the African Region) owing to these logistical problems. The World Health Organization (WHO) identified delays in detection as one of the major challenges facing the polio eradication programme^10^.

The DRC comprises 2,345,000 km² but has just one WHO-accredited laboratory, at the Institut National de Recherche Biomédicale (INRB) in Kinshasa, that is responsible for country-wide biological diagnosis of poliovirus. The INRB uses a sensitive and standardized WHO detection protocol that combines cell culture with intratypic differentiation (ITD) quantitative PCR (qPCR). Sequencing of the poliovirus VP1 capsid region is carried out at a separate laboratory in the Republic of South Africa, with a VP1 sequence required to both confirm poliovirus detection, or cases and to distinguish cVDPV from vaccine strains.

The GPEI is currently considering which approaches to use to achieve polio eradication in the last two WPV-endemic countries, Afghanistan and Pakistan, and to combat outbreaks of cVDPV in four of WHO’s six geographical regions^3^. The WHO Polio Eradication Strategy 2022–2026 (ref. ^3^) committed to improvements in detection and response. This includes direct detection of poliovirus in stool samples, thereby removing the need for the cell-culture-based detection algorithm according to the worldwide poliovirus containment aims^11^, and shifting of poliovirus testing and sequence analysis to country level.

These improvements in detection and response could be achieved by implementation of a direct molecular detection and nanopore sequencing (DDNS) method^12^. DDNS combines fast, direct detection from stool samples with on-site sequencing, avoiding international transport of samples and enabling quick response to outbreaks^9^. It could be implemented in any laboratory already using PCR, including INRB, in which Illumina and nanopore sequencing have been used for Ebolavirus, measles, monkeypox and severe acute respiratory syndrome coronavirus 2 (refs. ^13,14^).

In this Article, to validate DDNS implementation so that it can be considered as a recommended method by the WHO Global Polio Laboratory Network (GPLN)^15^, we undertook a prospective study in the DRC to evaluate application of the DDNS protocol and compared it with gold standard cell-culture methods for poliovirus surveillance. Here we report the sensitivity and specificity of DDNS compared with cell culture, sequencing accuracy, time taken in the lab and associated cost data.

## Comparison of DDNS with gold standard poliovirus diagnostic

Stool samples were tested in parallel using both the DDNS and the gold standard assay. A total of 2,339 prospective stool samples, from 1,159 AFP cases (each yielding 1 or 2 samples) and 62 case contacts or community samples (each 1 sample), were processed using 26 nanopore sequencing runs in a 141 day period, averaging one sequencing run every 5.4 days. DDNS identified 62 samples (2.7% of total samples) as positive for poliovirus, with 36 cVDPV2 (1.58%), 5 Sabin serotype 1 (0.30%), 19 Sabin serotype 3 (0.90%) and 2 that contained serotypes 1 and 3 Sabin poliovirus (0.09 %) (Table 1). The gold standard assay identified polioviruses in 51 samples, of which 31 samples were serotype 2 VDPV (1.33%), 4 were Sabin serotype 1 (0.17%) and 16 were Sabin serotype 3 (0.68%).

**Table 1:** Poliovirus detection by DDNS and the standard cell-culture, ITD and Sanger sequencing algorithm. Bold figures indicate shared detections between the two methods

		DDNS				
		Sabin 1	VDPV2	Sabin 3	Sabin 1 + Sabin 3	Negative
Cell culture, ITD and Sanger sequencing	Sabin 1	**3**	0	0	0	1
	VDPV2	0	**27**	0	0	4
	Sabin 3	0	0	**15**	0	1
	Negative	2	9	4	2	**2,271**

The sensitivity and specificity of detection for each poliovirus type for DDNS or the current gold standard assay is presented in Table 2. cVDPV2 detected by either method were not contamination because sequences differed from those of other samples (as shown in Supplementary Fig. 1). The sensitivity and specificity of the two methods did not differ significantly (Fisher’s exact test).

**Table 2:** Sensitivity and specificity by sample for detection of Sabin 1 and Sabin 3 polioviruses and VDPV2 by the standard cell-culture algorithm versus DDNS and vice versa. *P* values were generated using a two-sided Fisher’s exact test

		Culture versus DDNS (95% CI, *n*/*N*)	DDNS versus culture (95% CI, *n*/*N*)	Test for difference between methods, *P* value
Sabin 1	Sensitivity	43 (10–82, 3/7)	75 (19–99, 3/4)	0.55
	Specificity	100 (100–100, 2,331/2,332)	100 (100–100, 2,331/2,335)	0.37
VDPV2	Sensitivity	75 (58–88, 27/36)	87 (70–96, 27/31)	0.24
	Specificity	100 (100–100, 2,299/2,303)	100 (99–100, 2,299/2,308)	0.27
Sabin 3	Sensitivity	71 (48–89, 15/21)	94 (70–100, 15/16)	0.11
	Specificity	100 (100–100, 2,317/2,318)	100 (99–100, 2,317/2,323)	0.12

Two stool samples were available for 1,118 AFP cases, with 37 cases positive for poliovirus by either method. Eighteen cases had full concordance between both methods with both samples testing positive (Supplementary Table 1). There were no cases where both samples tested positive by the gold standard assay and yielded no positive DDNS result, whereas in nine cases with positive DDNS results no poliovirus was detected by the gold standard assay.

A single sample or pair of samples were available for 1,159 AFP cases. The sensitivity and specificity of detection were calculated for each AFP case (Table 3), and for only AFP cases where two stools were available (*n* = 1,118 cases; Supplementary Table 2).

**Table 3:** Sensitivity and specificity by AFP case for detection of Sabin 1 and Sabin 3 polioviruses and VDPV2 by the standard cell-culture algorithm versus DDNS and vice versa. *P* values were generated using a two-sided Fisher’s exact test

		Culture versus DDNS (95% CI, *n*/*N*)	DDNS versus culture (95% CI, *n*/*N*)	Test for difference between methods, *P* value
Sabin 1	Sensitivity	50 (7–93, 2/4)	100 (22–100, 2/2)	0.47
	Specificity	100 (100–100, 1,155/1,155)	100 (99–100, 1,155/1,157)	0.50
VDPV2	Sensitivity	70 (46–88, 14/20)	88 (62–98, 14/16)	0.26
	Specificity	100 (99–100, 1,137/1,139)	99 (99–100, 1,137/1,143)	0.29
Sabin 3	Sensitivity	75 (43–95, 9/12)	90 (55–100, 9/10)	0.59
	Specificity	100 (100–100, 1,146/1,147)	100 (99–100, 1,146/1,149)	0.62

## Time taken to confirm poliovirus by VP1 sequencing

**Fig. 1:**
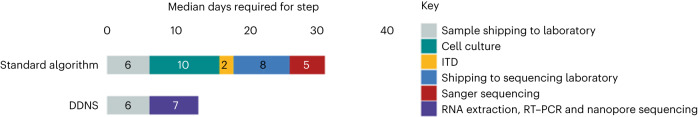
Median time required for each diagnostic step in the two protocols for 27 cVDPV2 positive stool samples. Time taken between case onset and a sequence being generated via DDNS and the standard algorithm were compared using a two-sided Mann–Whitney *U* test. Source data

During this study period, 27 samples containing VDPV2 had the VP1 region sequenced using both diagnostic methods. Only samples of programmatic importance (where vaccination response may be required; all serotype 2 viruses and any suspected vaccine-derived and wild-type polioviruses) are sequenced following cell culture whereas DDNS produces a sequence for positive samples without requiring additional sequencing elsewhere. For these 27 samples a median of 6 days was required between case onset and sample collection (range 2–21 days) and a further median 6 days was required between sample collection and arrival of samples at the sequencing laboratory (range 2–27 days). The time from receipt in the sequencing laboratory to a VP1 sequence took a median of 30 days (range 21–41 days) via the standard algorithm, including a median of 8 days (range 4–22 days) required for shipment between the virus isolation and sequencing lab, while DDNS was significantly quicker (*P* < 0.001, Mann–Whitney *U* test) requiring a median of 7 days (range 4–23 days) (Fig. 1).

## cVDPV2 outbreaks during the study period

**Fig. 2:**
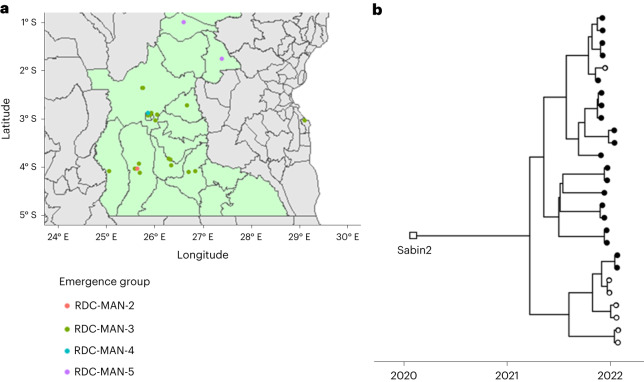
cVDPV2 outbreaks detected by DDNS. **a**, Cases from the four Maniema lineages detected during the study period (Maniema Province highlighted in green). Cases are plotted by district, with placement within the district determined at random. **b**, Tip-dated phylogenetic tree showing the maximum likelihood emergence date of RDC-Man-3 lineage and its subsequent diversification over time. Solid tips indicate that the DDNS detection was matched by a cell-culture-based detection of a cVDPV2 from the same sample. Cases confirmed by Sanger sequencing but without a corresponding DDNS sequence (*n* = 2) were not included in the analysis. Source data

During the study period, four cVDPV2 outbreaks occurred in the Province of Maniema in the DRC and confirmed through the routine gold standard algorithm. For two of the linages (RDC-MAN-3 and RDC-MAN-4) the sample confirming circulation (second case) was collected during the study period, while for RDC-MAN-2 the confirming sample was collected 42 days before the study period and processed during training. The gold standard detection algorithm required 27, 35 and 64 days, respectively (mean 42 days), to process these samples from collection to Sanger sequencing. These same samples were processed in 6, 20 and 30 days, respectively, by DDNS, despite the samples for RDC-MAN-2 being collected before the study period, a mean of 23 days quicker. The fourth outbreak lineage, RDC-MAN-5, only had the first positive sample collected during the study period, but this sample similarly yielded a VP1 sequence 29 days earlier by DDNS. The geographic spread of cases identified by DDNS for the four outbreaks and relatedness of RDC-MAN-3 outbreak lineage is shown in Fig. 2. Based on the poliovirus VP1 molecular clock and these DDNS-derived VP1 sequences we estimate that the RDC-MAN-3 lineage emerged from a OPV2 vaccination campaign performed in the first quarter of 2020 (mean date 26 January 2020, 95% highest posterior density 4 April 2019 to 16 September 2020).

## Comparison of sequences using DDNS and Sanger sequencing

Where consensus cVDPV2 VP1 sequences were available from both DDNS and Sanger sequencing of culture isolate for the same sample, the similarity of the sequences was determined. The mean VP1 sequence identity comparing DDNS and the gold standard algorithm (including Sanger sequencing) for the 27 cVDPV2 with results for both methods was 99.98% (range 99.60–100%). The absolute number of differences between sequences is presented in Table 4.

**Table 4:** Nucleotide differences in the VP1 region (903 base pairs) between sequences generated by Sanger sequencing of culture isolate and by DDNS from the same stool sample

Number of nucleotide differences	Count (%)
0	25 (92.6%)
1	1 (3.7%)
2	0 (0%)
3	0 (0%)
4	1 (3.7%)
5+	0 (0%)

## Cost and staffing for DDNS and gold standard assay

The DDNS assay consumable costs are approximately US$20 per sample when performing multiplexed sequencing runs of 96 samples or $25 per sample when performing runs of 45 samples in lower-throughput laboratories (Supplementary Table 3). These figures include chloroform treatment, RNA extraction, nested PCR and nanopore sequencing. For chloroform treatment, cell culture and qPCR alone, the cell-culture-based detection algorithm costs approximately $31 (Supplementary Table 3), in addition to the cost of Sanger sequencing and shipping to the National Institute for Communicable Diseases (NICD) in South Africa where the sequencing is performed. While some large items of equipment are required by both methods, DDNS avoids the requirements of microscopes, incubators, cell counters and tissue culture cabinets, while only needing the addition of a MinION or GridION sequencer (with MinIONs typically costing $1,000, including a sequencing reagents kit and a flow cell). Staff and facilities costs have not been included in the figures, yet the performance of DDNS at INRB required only five staff members; three laboratory scientists for RNA extraction, nested reverse transcription (RT)–PCR and nanopore sequencing and two bioinformaticians/data managers to perform data quality control and match the sequences to case data. Comparable steps from the cell-culture-based algorithm requires four laboratory scientists for cell culture and qPCR, two support staff for maintaining the facilities and the support of additional sequencing staff and bioinformaticians at the NICD.

## Discussion

Here we report a prospective validation study that compared DDNS with the gold standard assay for detection of poliovirus in stool samples in DRC. Our data confirm that DDNS can be applied as a rapid, sensitive and cost-effective tool for surveillance in the DRC. Our results demonstrate that it is feasible to implement DDNS in a national poliovirus laboratory. Although not statistically powered for a direct comparison with the gold standard assay, our study indicates that DDNS is at least as sensitive as culture for the detection of poliovirus.

Implementation of DDNS in this study shows that it detects poliovirus faster than cell culture. DDNS VP1 sequences for VDPV2 positive samples were on average generated a median 14 days after stool collection. This is similar to, but slightly slower than, the 12 days we predicted when estimating the performance of DDNS based on stool sample collections from 2016 to 2020 (ref. ^9^). Our earlier estimates did not account for sample batching to maximize efficiency and minimize costs. Further improvements in speed could be achieved by automated RNA extraction, or by decreasing sample delivery time to the laboratory, perhaps by drones given poor road conditions^16^, or through the establishment of additional regional laboratories within DRC.

For samples required to confirm three of the four cVDPV2 lineages, DDNS generated the VP1 sequence required to initiate a response a mean of 23 days (range 6–30 days) quicker than culture. Earlier detection and response to outbreaks leads to few cases and a higher probability of truncating ongoing transmission^2,9^. Despite the lower raw read sequencing accuracy of nanopore compared with Sanger sequencing, the generation of consensus sequences gave a mean similarity of 99.98%. The sequence identity was <100% for just 2/27 samples. Where a relatively large difference was observed (the sample with four nucleotide differences between consensus sequences from DDNS and Sanger sequencing) this may represent different viral populations within the gut of the AFP case. This case had a pair of stool samples collected a day apart, and the Sanger sequences for the pair also differed by one nucleotide. Through removal of competitive viral cell culture and with the use of next-generation sequencing, DDNS does however allow the identification of multiple viral templates from a single sample, as demonstrated by the detection of both Sabin 1 and Sabin 3 in two of the samples. Improved calling of haplotypes could even allow the resolution of very closely related viral populations (differing by only one or two nucleotides) from within a single sample.

The additional poliovirus detections by DDNS were not likely to be due to contamination, given that they tended to occur either in sample pairs from the same AFP case, or in sample pairs where culture tested positive for one of the pair. Furthermore, DDNS enables rapid identification of contamination with viruses of programmatic importance (wild type and VDPVs) because of the low likelihood of identical VP1 sequences apart from for those samples collected from the same case or their contact. A quality assurance programme for DDNS has been developed, including the use of a lyophilized virus positive control, and quality control flags built into bespoke software now developed for DDNS (PIRANHA^17^).

Implementation of the method does not require a cell-culture facility or the transfer of samples to an overseas laboratory for Sanger sequencing if local capacity is not available, allowing all steps to be performed at one site in a single streamlined workflow. The per sample cost is at least $10 lower for high-throughput laboratories that can maximize the benefit of sample multiplexing during sequencing. This excludes further savings from international shipment of samples and sequencing at a centralized hub. While staff salaries and facilities costs were not included in these calculations (and will vary greatly between countries), routine DDNS at INRB was implemented with contributions from five staff, compared to six for the cell-culture method. Moreover, it supported a workforce trained in molecular techniques including the preparation of sequencing libraries, performance of sequencing and the analysis of sequencing data. The skills and facilities required for DDNS can be rapidly redeployed to other pathogens during public health emergencies. With the global expansion of sequencing capacity there are increasing opportunities to foster the development of these skills and facilitate their contribution to disease surveillance and pathogen genomics, potentially through centralized bioinformatic support from either the GPLN, from sub-regional labs with expertise (for example INRB) or from other regional bodies (for example Africa CDC’s Pathogen Genomics Initiative).

One advantage of DDNS is the potential to completely replace cell culture in most polio laboratories, which is both costly and undesirable as poliovirus goes in to global containment^18^. For DDNS to be sustained, challenges with supply chains must be overcome, as countries likely to benefit most from rapid detection are also likely to be more difficult to supply with reagents for nanopore sequencing. A full economic costing for implementation across the GPLN would also be necessary before implementation. For laboratories that also test environmental surveillance (ES) samples, DDNS can be used for these samples, providing sequencing reads for multiple virus templates, as may occur in sewage^12^. However, direct detection methods typically only allow relatively small sampling volumes (hundreds of microlitres for an RNA extraction as opposed to 4 mL for the eight cell-culture flasks now employed); hence, greater concentration of ES samples and/or large volume RNA extractions will be required to allow achieving a similar or greater degree of detection sensitivity. We are currently optimising these methods for ES samples, which show considerable promise^19^.

During this research study, sequences were not used to inform outbreak response because the method has not yet been accepted or recommended by the GPLN^15^. We are now working to meet the requirements for GPLN recommendation, including pilot implementation of DDNS in additional laboratories worldwide. An additional method of direct detection by qPCR^20^ (without sequencing) is being evaluated by the GPEI, and a comparison between this method and DDNS has not yet been made. Further evaluation will be required to compare accuracy of detection and the speed at which a VP1 sequence can be generated by the two methods, along with consideration of ease of implementation and staff training requirements.

## Methods

### Sample collection

Stool samples were collected during routine AFP surveillance in DRC between 10 August 2021 and 4 February 2022. These diagnostic specimens were collected as part of the DRC Ministry of Health’s routine public health disease surveillance and polio eradication programme, and therefore consent for sample collection was waived. All preparation of samples for sequencing, genomic analysis and data analysis was performed on anonymized samples identifiable only by their laboratory or the epidemiological identifier. All relevant ethical regulations were followed.

All 2,339 samples received at the national polio laboratory (INRB) from AFP cases, the community and contacts were processed in this study. It is recommended that two stool samples are collected from children aged 0–14 years old with AFP, within 14 days of onset of paralysis and at least 24 h apart. Single stool samples from healthy contacts of children with AFP are additionally typically collected from children aged <5 years old and occasionally from the wider community.

### Sample processing for cell culture

Chloroform-treated stool supernatant was prepared and processed as described in ref. ^21^ as part of routine poliovirus stool surveillance. Briefly, 0.8 ml of chloroform-treated stool supernatant was inoculated into two culture flasks containing L20b cells and two flasks containing RD cells. After two passages, 1 μl of the supernatant of culture flasks where the cells had shown suspected poliovirus cytotoxic effects on L20B was used for intertypic qPCR differentiation using the Poliovirus rRT-PCR ITD v5.2 kit following the manufacturer’s protocol with routine further investigation of VDPVs.

Where sequencing was required, the isolate culture was dried on Flinders Technology Associates cards and shipped to NICD in South Africa for Sanger sequencing.

### RNA extraction for DDNS

Chloroform-treated stool supernatant underwent RNA extraction using either QIAamp Viral RNA Mini Kits (#51106, used for samples processed August 2021 to September 2021) or Roche High Pure Viral RNA Kits (#11858882001, used for samples processed October 2021 to February 2022) according to manufacturers’ protocols using a volume of 140 µl and 200 µl of supernatant, respectively. RNA extraction kit selection was determined by kit availability, but both kits have been validated for the DDNS method^22^. Extracted RNA was stored at +4 °C during the preparation of the nested PCR if performed on the same day or −80 °C if delayed more than 24 h.

### DDNS

DDNS based on a nested, barcoded PCR and amplicon sequencing on nanopore sequencers was performed as described in ref. ^12^. In brief, a nested PCR was performed using 5 µl of extracted RNA and pan-Enterovirus primers^23^ with the product used for a poliovirus specific VP1 PCR using barcoded primers^12^. Two microlitres of each PCR product was pooled before cleaning with AMPure XP beads (Beckman Coulter, #A63880) and the sequencing library prepared with Oxford Nanopore Technologies LSK-110 ligation sequencing kits. The complete protocol can be found in ref. ^24^.

Libraries were sequenced on the Oxford Nanopore Technologies GridION, MK1c or MinION sequencers for between 4 h and 12 h using R9.4 flow cells. Sequencing runs were performed between 29 September 2021 and 17 February 2022. The study team performing DDNS were unaware of results from the gold standard algorithm while samples were processed.

### Bioinformatics

Sequence basecalling was performed using guppy with demultiplexing and mapping of the reads performed using RAMPART^25,26^ and the realtime-polio analysis module^25^. VP1 consensus sequences were generated by four iterative rounds of mapping using the mafft algorithm^27^ and polishing with racon^28^ before consensus calling with medaka^29^. The dated maximum-likelihood tree for the RDC-MAN-3 outbreak was created in R version 4.1.3 using the Analyses of Phylogenetics and Evolution (ape) package^30^, Phylogenetic Reconstruction and Analysis (phangorn) package^31^ and the treedater package^32^. A molecular clock rate of 0.01 substitutions per site per year was assumed on the basis of ref. ^33^.

### Statistical Analysis

Time taken between case onset and a sequence being generated via DDNS and the standard algorithm were compared using a two-sided Mann–Whitney *U* test. Exact binomial confidence intervals were calculated for the sensitivity and specificity of the two methods and comparison made using a two-sided Fisher’s exact test.

### Reporting summary

Further information on research design is available in the Nature Portfolio Reporting Summary linked to this article.

## Supplementary information

Supplementary Information Supplementary Tables 1–3 and Fig. 1.

Reporting Summary

## Data Availability

Sequences generated during this study are available at the European Nucleotide Archive under accession number PRJEB61181. Sample metadata are shown in Source Data Fig. 2. Source data are provided with this paper.

## Source data

Source Data Fig. 1 Table of time required for each step in each method.
Source Data Fig. 2 Descriptors of DDNS sequences.

## Code availability

The current version of the analytical software for analysing DDNS data can be found at https://github.com/polio-nanopore/piranha.
