# Supplementary material for: Sensitive poliovirus detection using nested PCR and nanopore sequencing: a prospective validation study
Source: Nat Microbiol. 2023 Aug 17;8(9):1634–40. doi: 10.1038/s41564-023-01453-4 (PMC10465353; doi:10.1038/s41564-023-01453-4)
Supplement: Supplementary file 1 — Supplementary Tables 1–3 and Fig. 1. [file 41564_2023_1453_MOESM1_ESM.pdf]

nature microbiology

Article

https://doi.org/10.1038/s41564-023-01453-4

Sensitive poliovirus detection using nested PCR and nanopore sequencing: a prospective validation study

In the format provided by the authors and unedited

Supplemental Table 1

		DDNS		
		+ +	+ -	- -
**Culture**	+ +	**18**	0	0
	+ -	3	**4**	3
	- -	4	5	**1081**

Supplemental Table 1 – *Concordance of the cell-culture algorithm and DDNS for the testing of paired stool samples. Bold figures indicate shared detections between the two methods.*

Supplemental Table 2

		Culture vs DDNS (95% CI, n/N)	DDNS vs culture (95% CI, n/N)	Test for difference between methods, P-value
**Sabin 1**	Sensitivity	50 (7-93, 2/4)	100 (22-100, 2/2)	0.47
	Specificity	100 (100-100, 1114/1114)	100 (99-100, 1114/1116)	0.50
**VDPV2**	Sensitivity	70 (46-88, 14/20)	88 (62-98, 14/16)	0.26
	Specificity	100 (99-100, 1096/1098)	99 (99-100, 1096/1102)	0.29
**Sabin 3**	Sensitivity	75 (43-95, 9/12)	90 (55-100, 9/10)	0.59
	Specificity	100 (100-100, 1105/1106)	100 (99-100, 1105/1108)	0.62

Supplemental Table 2 - *Sensitivity and specificity by AFP case where two samples are available for detection of Sabin 1 and Sabin 3 polioviruses and VDPV2 by the standard cell-culture algorithm versus DDNS and vice versa.*

Supplemental Table 3

Chloroform treatment - required for both methods	Cost in Dollars	Comments
Chloroform (1mL per sample)	$0.08	
Glass beads 3mm diameter (1g per sample)	$0.70	
PBS (10 mL per sample)	$0.60	
50 mL centrifugre tubes (1 persample)	$0.60	
Total	$1.99	

Cell-culture and ITD - Standard algorithm	Cost in Dollars	Comments
Culture flasks (12 per sample)	$17.05	
Eagle's minimum essential medium (10 mL per flask)	$3.67	
L-glutamine 200 mM (0.11 mL per flask)	$0.36	
Fetal calf serum (0.22 mL per flask)	$4.60	
HEPES 1M (0.11 mL per flask)	$0.92	
Strippettes	$0.62	
RNA extraction kit	$0.65	Costed for MagMAX viral RNA Isolation kit (AM1939)
Pipette tips	$1.52	
qPCR plates	$0.04	
Primers and probes	$0.01	
qPCR ToughMix	$0.43	Assuming 10 % of samples are positive by cell-culture
Total	$29.86	

DDNS - High workload (90 samples per flow cell)	Cost in Dollars	Comments
RNA extraction kit	$6.47	Costed for MagMAX viral RNA kit
Superscript III One-Step RT-PCR System	$4.45	200 extractions per kit
DreamTaq	$0.46	
NEBNext Companion Module	$0.51	24 runs per kit
MinION Flowcell R9.4.1	$2.28	Assuming 2 runs per flow cell
Flowcell wash kit	$0.45	
Ligation Sequencing kit	$1.64	Assuming 90 samples per run and 6 sequencing runs per kit
Primers	$0.01	Assuming 90 samples per sequencing run
Pipette tips	$1.69	Assuming 90 samples per sequencing run
PCR plates	$0.13	Assuming 90 samples per sequencing run
Total	$18.08	

DDNS - Low workload (45 samples per flow cell)	Cost in Dollars	Comments
RNA extraction kit	$6.47	Costed for MagMAX viral RNA kit
Superscript III One-Step RT-PCR System	$4.45	
DreamTaq	$0.46	
NEBNext Companion Module	$1.01	
MinION Flowcell R9.4.1	$4.56	Assuming 45 samples per sequencing run
Flowcell wash kit	$0.90	Assuming 45 samples per sequencing run
Ligation Sequencing kit	$3.27	Assuming 45 samples per sequencing run
Primers	$0.01	Assuming 45 samples per sequencing run
Pipette tips	$1.69	
PCR plates	$0.13	
Total	$22.95	

Total per sample cost	
$31.85	Cell-culture and ITD
$20.07	DDNS (High load)
$24.94	DDNS (Low load)

Supplemental Table 3 - *Costings of reagents for the cell-culture and AITD algorithm, and for DDS in laboratories with high and low sample throughput. Reagents required for the cell-culture and ITD protocol have been estimated given the guidance in the World Health Organisation Polio Laboratory Manual, 4th edition, 2004. Reagents required for DDNS have been calculated according to the protocol presented on dx.doi.org/10.17504/protocols.io.81wgbpkmovpk/v3. Laboratories classified as having 'high' loads are ones able to assemble near-complete sequencing runs without waiting for over 1 week for samples to accumulate. Laboratories classified as having 'low' loads would be unable to*

fully maximise the benefit of multiplexing and would instead perform sequencing runs below their maximum capacity; in this case assuming testing of only 45 samples a week.

Supplemental Figure 1

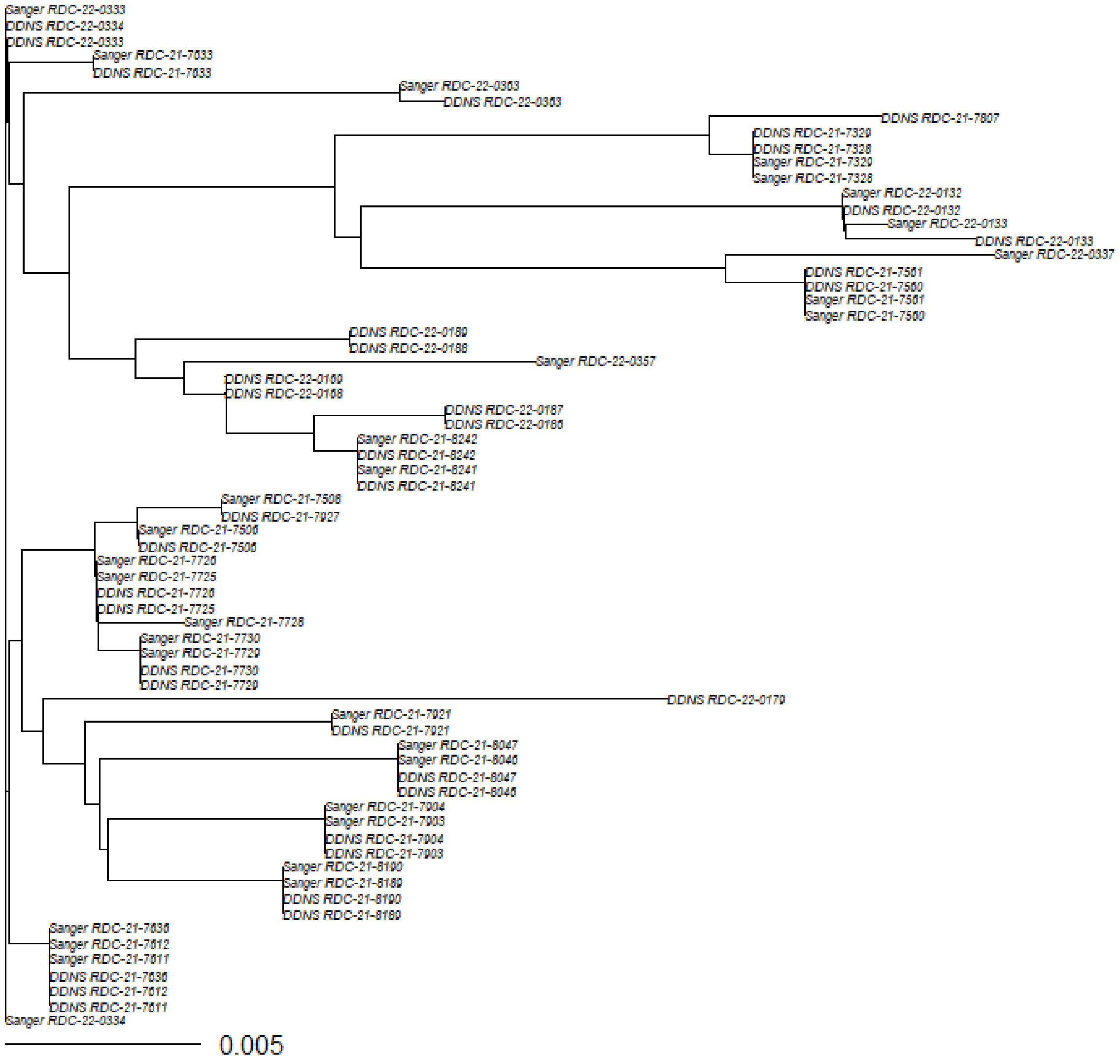

Supplemental Figure 1 – Relatedness of all VDPV2 sequences detected by either DDNS or the cell-culture and Sanger sequencing during the study period. The distance between samples was calculated using the ape package in R, using the TN93 evolutionary model.
